# Chronic tarsal conjunctivitis

**DOI:** 10.1186/s12886-016-0294-1

**Published:** 2016-07-29

**Authors:** Nicholas Cook, Fizza Mushtaq, Christina Leitner, Andrew Ilchyshyn, George T. Smith, Ian A. Cree

**Affiliations:** 1 Primary Care Ophthalmology Service, Central Surgery Eye Clinic, Rugby, CV21 3SP UK; 2 Department of Ophthalmology, Leicester Royal Infirmary, Leicester, LE1 5WW UK; 3 Departments of Ophthalmology, Dermatology, and Pathology, University Hospitals Coventry and Warwickshire, Coventry, CV2 2DX UK; 4 Tasmanian Eye Clinics, 2-4 Kirksway Place, Hobart, TAS 7000 Australia

**Keywords:** Conjunctivitis, Contact allergy, Cosmetic, Epiphora, Steroid, Allergen

## Abstract

**Background:**

Toxicity is rarely considered in the differential diagnosis of conjunctivitis, but we present here a new form of toxic conjunctivitis with unusual clinical features. Between 2010 and 2013, a new clinical presentation of chronic conjunctivitis unresponsive to normal treatment was noted within a Primary Care Ophthalmology Service.

**Methods:**

Retrospective review of case records and histopathology results.

**Results:**

A total of 55 adult patients, all females, presented with epiphora and stickiness. They did not complain of itch and had had symptoms for an average of 9 months. Clinical examination showed bilateral moderate to severe upper and lower tarsal conjunctival papillary reaction, without corneal or eyelid changes and mild bulbar conjunctival hyperaemia in a third of cases. Biopsies were taken in 15 cases to exclude an atypical infection or lymphoma. Histologically, there was a variable superficial stromal lymphocytic infiltrate, involving the epithelium in more severe cases. The majority of the cells were CD3 positive T-lymphocytes and follicle formation was not noted. The clinical history in all cases included prolonged use of eye make- up and other facial cosmetic products. Clinical symptoms of epiphora settled with topical steroid drops, but the clinical signs of chronic tarsal inflammation persisted until withdrawal of the facial wipes thought to contain the inciting agent, though the exact nature of this remains unclear.

**Conclusion:**

The presentation, appearances, histological features are consistent with a contact allergen-driven chronic conjunctivitis. Steroid treatment provided good relief of symptoms and patients were advised to avoid potential contact allergens. Management remains difficult. Further research into contact allergies of mucous membranes and identification of its allergens is required.

**Electronic supplementary material:**

The online version of this article (doi:10.1186/s12886-016-0294-1) contains supplementary material, which is available to authorized users.

## Background

Many forms of chronic conjunctivitis are recognised. The commonest type is probably chronic allergic conjunctivitis manifesting as itchy red eyes with thickened eyelid margins and associated periorbital dermatitis, usually associated with atopy [1, 2]. Itch is always a major feature of chronic allergic conjunctivitis. Other forms of chronic conjunctivitis include meibomian gland disease blepharo-conjunctivitis, contact lens-related giant papillary conjunctivitis, floppy eyelid syndrome, post chemotherapy conjunctivitis, cicatrising conjunctivitis, periocular dermatitis, giant fornix syndrome, and chlamydial conjunctivitis [3]. Toxicity is rarely considered, and there are relatively few publications in the field, but chronic toxic conjunctivitis has been described presenting with a clinical picture of watery discharge, conjunctival papillary initial reaction, follicular subsequent reaction, often eyelid dermatitis and inferior punctal erosions [4–6].

We have recently noted increased referral rates to our Primary Care Ophthalmology (PCO) service of patients with an atypical presentation with epiphora as the primary symptom. Other common causes of epiphora include actual nasolacrimal duct obstruction and functional nasolacrimal duct obstruction, but we have not noted any increase in the numbers of patients presenting with either condition. Between 2010 and 2013, one of us (NC) noted this new clinical presentation of chronic conjunctivitis, and in 2013, this became epidemic with 17 new cases referred in 2013 and 29 in the first 8 months of 2014. This paper presents a retrospective review of the clinical features of a series of 55 cases of this new form of conjunctivitis, to September 2014.

## Methods

Retrospective review of standard clinical and laboratory investigations was conducted in a series of 55 patients with a newly recognised form of conjunctivitis. Our review conforms to the tenets of the Declaration of Helsinki. Due to its retrospective nature, UK Health Research Authority Ethics Committee approval was not required. Consent for publication is not required as long as the patients are not identifiable. The patients undergoing biopsy were fully informed of the risks and benefits involved and gave written informed consent for the procedure, including use of ocular photographs, tissue, and related data for research and teaching.

### Clinical management

Standard slit lamp examination with tarsal conjunctival digital photography (Topcon 3D OCT 2000) was performed to document conjunctival changes. In the early cases some patients underwent sac washout to determine nasolacrimal duct patency, but this test was rapidly considered irrelevant since the primary site of the problem is the conjunctiva and not the punctum or nasolacrimal duct. The only effective treatment was topical steroid drops. Consequently careful follow up was required particularly to monitor intraocular pressure. Resolution of the condition was considered when the patient had been off topical steroids for 2 months, with no epiphora symptom and normal tarsal conjunctival appearances.

### Microbiology

In four patients swabs were sent for chlamydial testing (BD Viper, Becton Dickinson, Oxford, England), all of which were negative. No swabs were sent for bacteriological analysis.

### Biopsy and histopathology

Since the course of the condition was prolonged and aetiology uncertain, fifteen newly presenting consecutive patients consented to conjunctival biopsy. Punch biopsies (1 mm) were taken from the tarsal conjunctiva in 15 cases. These were fixed in neutral buffered formalin (4 % formaldehyde), processed and embedded in paraffin wax. Sections cut at 4 μm were stained with haematoxylin and eosin, and examined by direct microscopy. Immunohistochemistry for CD3 (a T-lymphocyte marker) and CD20 (a B-lymphocyte marker) was performed to phenotype lymphocytes within the infiltrate.

### Allergen sensitivity testing

Skin patch-testing with IQ Ultra (Chemotechnique Diagnostics, Sweden) was performed in 43.6 % (24/55) cases by a dermatologist, according to manufacturer’s instructions. All patients were tested with the British standard battery (British Society for Cutaneous Allergy) including EDTA and benzalkonium chloride. Results were read on day 2 and day 4 following initial application. Erythema with infiltration, papules or vesicles on the skin were considered positive results. Their clinical relevance was interpreted in association with the history of present complaints, improvement of symptoms by withdrawal of a suspected contact allergen or previous reactions on exposure to contact allergens. Questionnaires and electronic hospital files were used in order to obtain information on a personal history of atopy (at least one of the following conditions should be present in one patient: asthma, eczema or hayfever), occupational exposure to contact allergens, the application of facial cosmetic products and exposure to airborne contact allergens.

## Results

### Clinical features

A total of 55 patients were included in this retrospective case series. All were adult females with a median age of 44 years (range 17–72) presenting during the period 2010–2014 as referrals from primary care practitioners in whom standard treatments for epiphora and conjunctivitis had already been undertaken and had not been successful. The clinical findings are summarised in Additional file 1: Table S1. Epiphora was the commonest presenting symptom. Associated symptoms included stickiness, and itch featured in only 1 patient. None wore contact lenses and all had had the condition for at least a month (mean 9 months, range 1 – 36 months). All patients had previously used cosmetics on a daily basis.

**Fig. 1:**
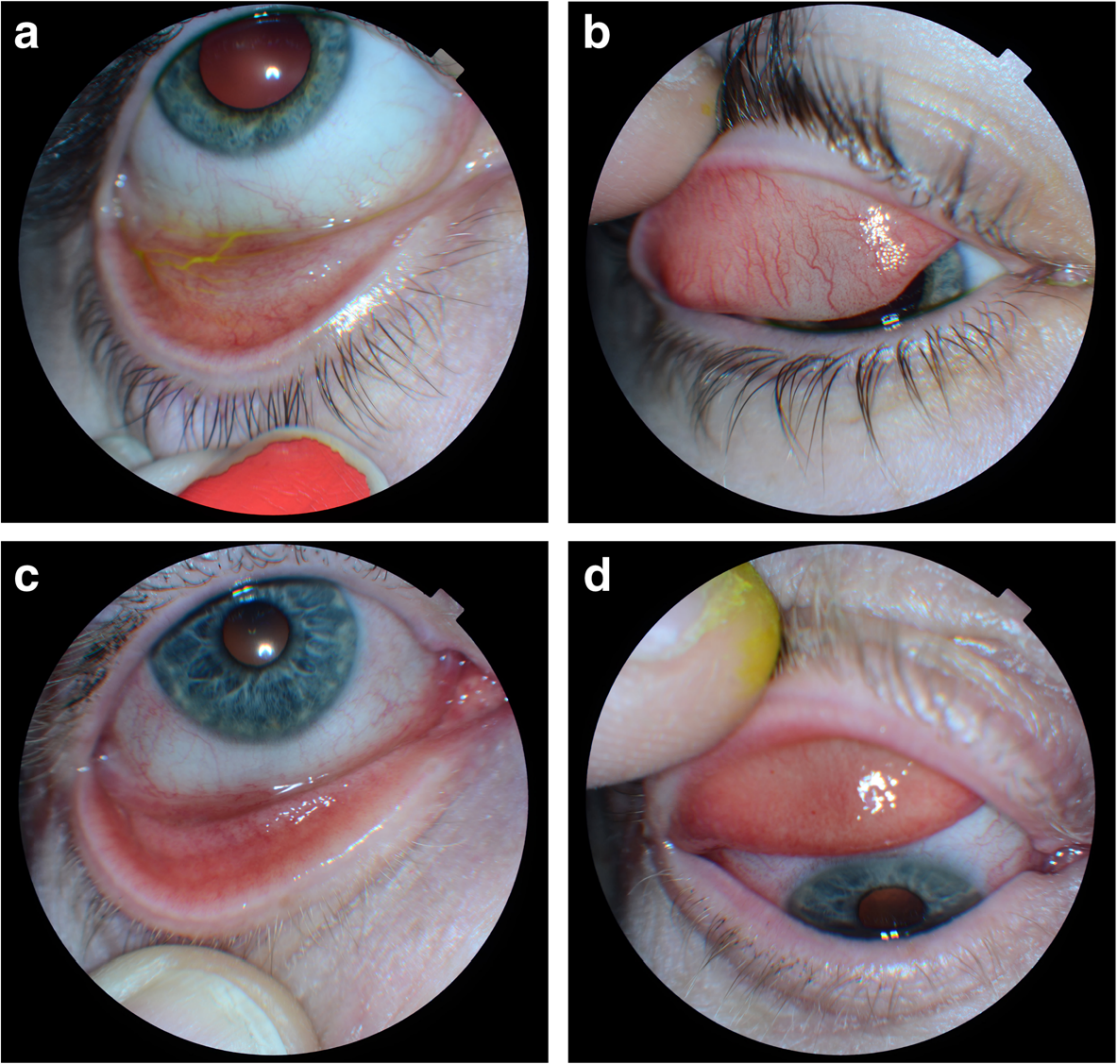
Clinical appearances of the eye in two representative cases, showing the superior and inferior tarsal conjunctival appearances in two patients. **a** Lower lid, **b** upper lid from one patient, and **c** lower lid and **d** upper lid from another. Both show typical papillary appearances with some hyperaemia

The cardinal clinical sign was a bilateral moderate to severe upper and lower tarsal conjunctival papillary reaction (Fig. 1). In a third of cases there was a mild bulbar conjunctival hyperaemia, but in most cases the bulbar conjunctiva was normal. Important negative signs included a lack of corneal changes, eyelid dermatitis or lid margin thickening. Differential diagnoses were excluded by history, examination or lack of response to treatment (including previous treatment prior to referral).

### Microbiology

In 7.1 % (4/55) patients, standard microbiology excluded chlamydial infection. Although not clinically a follicular conjunctivitis, all patients were treated empirically with a trial of 1 g azithromycin after counselling, with no response in either symptoms or signs.

### Histopathology

**Fig. 2:**
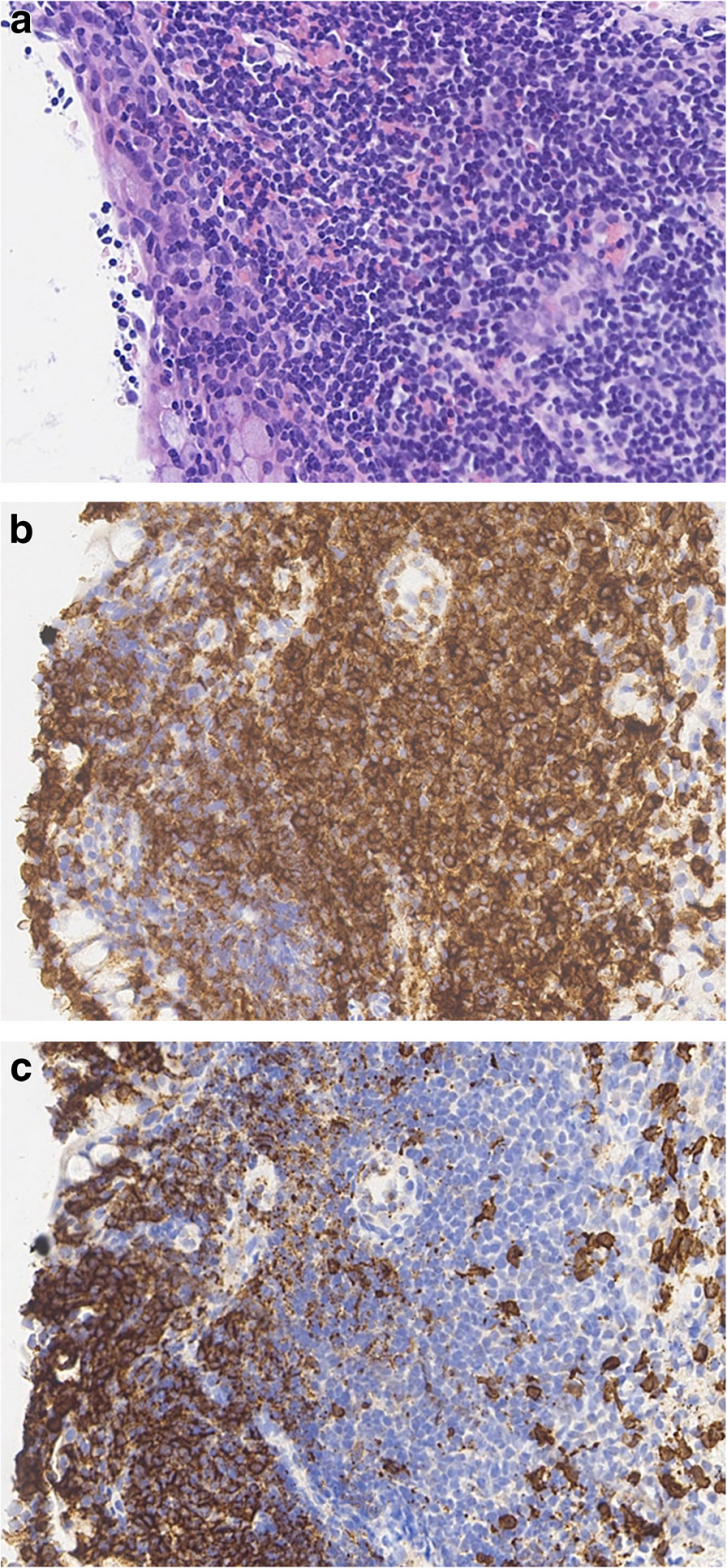
Histological appearance of the inflammatory infiltrate in the conjunctiva. **a** H&E showing involvement of the conjunctival stroma and epithelium by lymphocytic inflammation. **b** CD3 stained section showing that the infiltrate consists mainly of T lymphocytes. **c** CD20 stained consecutive section showing that there are also significant numbers of B lymphocytes

H&E slides showed conjunctival tissue with mild (*n* = 3), moderate (*n* = 5), or severe (*n* = 4) superficial stromal chronic inflammation, involving the epithelium in all severe and moderate cases. Inflammation could not be graded in 3 cases with insufficient material for this to be assessed. The inflammation was predominantly lymphocytic with a striking lack of lymphoid follicle formation (Fig. 2). Goblet cell numbers were variable, with both increased and decreased numbers noted. On immunohistochemistry, the majority of cells were clearly of CD3 positive T-lymphocytes, with variable but lower numbers of CD20 positive B-lymphocytes (Fig. 2). There was no evidence of lymphoma, cicatricial pemphigoid, vernal conjunctivitis, and other infectious causes. There were few neutrophils, eosinophils or macrophages and no granulomas were observed. No basal epithelial apoptosis were noted, excluding a lichenoid reaction. The appearances were thought to be consistent with contact allergy.

### Allergen sensitivity testing

In total, 72 % (40/55) were invited for patch-testing, 40 % (16/40) did not attend their appointment. Amongst all patients patch-tested, 45.8 % (11/24) were sensitised to Nickel, 4.1 % (1/24) to methylisothaizolinone (MI) (0.2 % strength), 4.1 % (1/24) to fragrance mix 1 and balsam of Peru, 4.1 % (1/24) to PPD (p-Paraphenylendiamine) and 4.1 % (1/24) to Potassium dichromate.

Only 9 % (1/11) of Nickel positive patients confirmed a past medical history of skin reactions to Nickel containing custom jewellery, no one else sensitised on patch-testing to one or more contact allergens had a relevant past medical history of contact dermatitis. 16.6 % (4/24) had a personal history of atopy. No one was exposed to potential contact allergens at work or during leisure time.

### Response to treatment

The first 15 patients in this series were given olopatadine (Opatanol) anti-histamine and nedocromil (Rapitil) for several months, with no response. Clinical symptoms of epiphora typically settled within a week or two of starting topical steroid drops, either dexamethasone 0.1 % (Maxidex) or prednisolone 0.5 % (Predsol), but the clinical signs of chronic tarsal inflammation usually remained for many months despite continuous topical steroid use. As the frequency of the topical steroid drops was reduced over months and the strength was reduced to fluorometholone, the symptom of epiphora and the signs of chronic tarsal inflammation tended to recur. This pattern of response to topical steroid and recurrence on stopping steroid persisted until the summer of 2014 when a contact sensitivity response was suspected. At this point all patients were advised to refrain (or at least be minimalist) about application of facial products and to refrain from using facial wipes. Since this approach was taken in 83.6 % (46/55) cases it is known that the condition is completely resolved, with all patients in this series discharged by April 2015. One of this series of patients developed raised intraocular pressure requiring cessation of steroid treatment. The reasons for loss to follow up, 16.3 % (9/55), is unknown. As access to the PCO service by self-referral is relatively straight forward, one reason for non-attendance might be complete resolution of symptoms.

In the initial stages, 8 patients were referred into the local Hospital Eye Service. Some of these patients subsequently returned to the Primary Care Ophthalmology service for continuity of care. Additional file 2: Table S2 describes the outcomes for these patients.

## Discussion

**Fig. 3:**
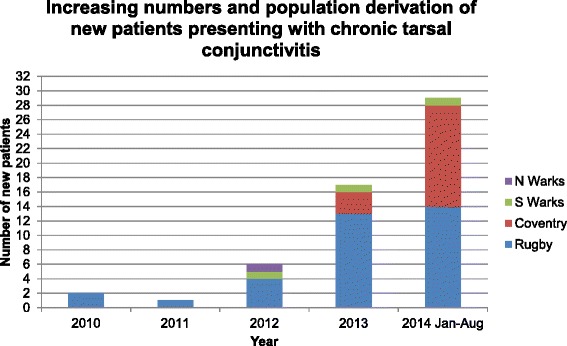
Increasing numbers and population derivation of chronic tarsal conjunctivitis over five years

We describe here a rapid rise in incidence of a new form of chronic conjunctivitis (Fig. 3), which we believe to be a form of contact conjunctivitis related to changes in the constituents of peri-ocular cosmetics or the facial wipes used to remove them. Cosmetics have been previously known to cause problems in the eye [7] and some toxicity testing in the eye is performed in most products on the market, using the Draize eye test and animal-free alternatives [8, 9]. This is usually aimed at excluding corneal toxicity, but in this instance it appears that effects on the conjunctiva have produced a clinically well-defined syndrome.

While this condition is likely to be present in the patient population presenting to hospital ophthalmology departments, it may be missed amongst the multitude of other cases. The Primary Care Ophthalmology service, staffed by a single practitioner general ophthalmologist, sees nearly all new patients with epiphora for the population of Rugby (*n* = 100,000) as well as patients from other nearby areas. Assuming a UK population of 63 million and that this new disease presentation is similarly represented through the UK, extrapolation suggests that up to 13,000 patients may have presented with this condition within the UK in 2014.

This form of conjunctivitis is of particular concern since the condition requires follow up and topical steroid treatment for months or even years unless the correct management is undertaken. The only effective treatment we have identified to date is topical steroid and avoidance of periocular preparations and facial wipes. Cataract formation is a risk when steroids are used for prolonged periods, and in most cases, the steroid was reduced from standard strength (eg dexamethasone 0.1 %) to weakest strength (eg Fluorometholone, FML) within 3 months. Being on a reducing dose of FML for 6–12 months probably does not have a high cataract progression risk. However, it is of concern that many of these patients are at risk of remaining on topical steroids for long periods to control their symptoms unless they are prepared to cease using all facial products and use of facial wipes and eliminate the cause.

In December 2013, there was extensive UK media coverage about an epidemic of dermatological problems being caused by the chemical methylisothaizolinone (MI) and methylchlorisothiazolinone (MCI), both collectively also known as Kathon, which are used to increase the shelf life of cosmetics, lotions, soaps, shampoos, other body products and skin cleansers [10, 11]. These were initially introduced as cosmetic preservatives in 2006 and since then have become widely used. Since their introduction unprecedented number of contact allergies and contact dermatitis have been reported. In the US the Environmental Working Groups Cosmetic Database considers MI to be a moderate health hazard because it is a chemical irritant that can affect the skin, eyes or lungs. MI has been banned in Canada, but is still popularly used in the US. MI has been considered as “contact allergen for 2013” by the American Contact Dermatitis Society. The European Commission Scientific Committee on Consumer safety (http://ec.europa.eu/health/scientific_committees/consumer_safety/docs/sccs_o_145.pdf) considers MI to be a strong sensitiser with a potency category of “extreme” and that the dramatic rise in rates of contact allergy to MI, as detected by diagnostic patch tests, is unprecedented in Europe. Due to the rising number of cases in 2014 and an awareness of potential problems with Kathon, patients were advised to try and avoid products containing Kathon, though the problem could certainly not be attributed to this agent. Skin patch testing was undertaken to identify possible causes, but the results did not support our suspicion. This might be simply due to failure in identifying the responsible contact allergen, which is known to be difficult. Random testing is not recommended as it creates a high rate of false positives [12] and atopic disease is associated with a higher risk of contact allergy [13]. In those patients who attended patch testing, withdrawal of suspected contact allergens did not show a clear trend of improvement of symptoms, likewise continuous exposure did not coincide with persistence of symptoms.

Sensitisation of patients to contact allergens on patch-testing did not seem to be linked to true contact dermatitis which is again in keeping with data on false positivity in the literature [14]. The high percentage of nickel sensitisation in our patients can be explained by our cohort being exclusively female [13]. The mechanisms of nickel hypersensitivity have been studied in detail, and are the result of haptenisation of proteins following skin exposure leading to the activation of hapten-specific T-lymphocytes [15]. The fact that women with positive test results did not have a history of contact dermatitis and equally, current symptoms of tarsal conjunctivitis do not seem to be associated with the sensitisation shown on testing, reflects the low sensitivity and specificity of 70 % of patch testing [14].

Cosmetic products such as eye shadows are known to contain nickel particles in variable concentrations. Brown and green colours contain the highest nickel levels and lead potentially to eye lid dermatitis and allergic contact conjunctivitis. Only one of our patients had a history of contact dermatitis to custom jewellery, a positive test to nickel but no symptoms on current continuous use of brown and green eye shadow. Overall, the skin testing results have to be interpreted with caution as the data are incomplete. Half of the patient cohort failed to attend testing and not every attendee completed a questionnaire.

The suitability of skin patch-testing for diagnosing contact dermatitis in mucous membranes is contentious. To our knowledge, no data are available on sensitisation thresholds of conjunctiva compared to the skin but there is literature raising awareness of different sensitisation thresholds of skin in different parts of the body such the back compared to the eyelids due to the variable thickness of the skin and the impact on penetration of contact allergens. This requires different concentrations of the contact allergen in order to achieve the same reaction [16]. The eye lashes and the tear film act as mechanical barrier to ocular exposure, but once these barriers are overcome, the highly vascularised conjunctiva facilitates access for contact allergens. One study by Villarreal reports the sensitivity of patch testing for allergic type 4 conjunctivitis as 74 % [17], which is similar to that for allergic contact dermatitis [14]. Its negative predictive value was low at 41 %. In 24 % of patients an additional conjunctival challenge had to be performed to diagnose an underlying contact allergy [17]. Unfortunately, in our series, the dermatology department undertaking patch-testing for this study was unable to perform conjunctival challenge tests.

Finally, the tarsal conjunctivitis could be an irritant conjunctivitis rather than a true type 4 mediated allergic conjunctivitis and therefore patch-testing failed to identify the culprit. The irritant threshold is proven to be different in skin and mucous membranes and recovery will be more difficult as withdrawal of the trigger does not result in rapid improvement as in contact allergic reactions. For instance, the irritant threshold for kathon on mucous membranes is lower than on the skin causing corrosion [18].

Our management (Table 1) was based on topical ocular steroid treatment with removal of facial products, including all facial wipes/make-up remover wipes, cosmetics, and moisturising products. The key attribute of the patients who have been discharged was a willingness to reduce significantly the products that they were putting on and around the eyelids. Some patients stopped everything, while others restricted application of facial products to 2–3 nights out a month. Clearly, it is important to establish the exact aetiology of this type of conjunctivitis.

**Table 1:** Management strategy for chronic tarsal conjunctivitis

1) Investigation by ophthalmology, including examination, swabs for chlamydia and biopsy (if necessary) to exclude other conditions.
2) Initiate treatment with a reasonable strength of topical steroid. Despite a rapid response of symptoms, continue on these drops for at least one month. Then slowly tail off over at least three months, titrating clinical features of tarsal conjunctival inflammation with strength and frequency of drops used. Don’t stop/tail off too quickly or symptoms and signs will recur.
3) Reduce as far as possible all facial products. This applies to facial wipes/make-up remover wipes/cosmetics/moisturising products.
4) On some occasions, the strength of topical steroid may have to be increased to control the symptoms when tarsal conjunctival inflammatory signs remain. Such resistance to treatment is to be expected if the underlying irritant is still being applied on and around the eyelids.
5) Elimination of the use of all products to the skin of eyes and eyelids is the best advice, but few women are prepared to consider this.

In some patients, the onset of symptoms appeared to coincide with the use of a supermarket brand of facial wipes. The brand of facial wipes concerned did not contain MI or MCI. On further questioning of the female patients as they have come back for review, at least 17 patients (c 30 % of cases presently under active review) were found to have used or be using these branded facial wipes. Those that were using facial wipes observed resolution of symptoms on ceasing using the facial wipes, in conjunction with several months of topical steroid drops. The vendor did change the formulation of their wipes from September 2015, and no new cases have been seen since December 2015. The main change in formulation was the supplier changing preservatives (to a paraben and phenoxyethanol free recipe). While there do not appear to be any published studies linking phenoxyethanel with conjunctivitis, parabens in eye-drops have been noted to cause contact dermatitis [19], and it is possible that this is the causative agent responsible for the condition we have observed.

## Conclusion

Chronic tarsal conjunctivitis is an unusual form of conjunctivitis that appears to be related to the use of a single brand of facial wipes, and may be toxic or contact-allergen driven by paraben used as a preservative. It is important that ophthalmologists recognise this new condition and, in conjunction with the patient, consider the management strategy detailed in this paper.

## Additional files

Additional file 1: Table S1. Clinical features of 55 patients with Chronic Tarsal Conjunctivitis. Inflammation was graded empirically on a scale from + (mild) to +++ (severe). (DOCX 24 kb) Additional file 2: Table S2. Outcome of 55 patients with Chronic Tarsal Conjunctivitis, as of April 2015. (DOCX 26 kb)
