# Supplementary material for: Chronic tarsal conjunctivitis
Source: BMC Ophthalmol. 2016 Jul 29;16:130. doi: 10.1186/s12886-016-0294-1 (PMC4965890; doi:10.1186/s12886-016-0294-1)
Supplement: Additional file 1: Table S1. — Clinical features of 55 patients with Chronic Tarsal Conjunctivitis. Inflammation was graded empirically on a scale from + (mild) to +++ (severe). (DOCX 24 kb) [file 12886_2016_294_MOESM1_ESM.docx]

**Table 1:** Clinical features of 55 patients with Chronic Tarsal Conjunctivitis. Inflammation was graded empirically on a scale from + (mild) to +++ (severe).

| **Patient ID** | **Clinical features**  (Yes/No) | | | | | | | | **Duration of symptoms at presentation (months)** | **Tarsal conjunctival biopsy** |
| --- | --- | --- | --- | --- | --- | --- | --- | --- | --- | --- |
|  | **Epiphora** | **Stickiness** | **Itch** | **Tarsal conjunctival papillary reaction** | **Bulbar conjunctiva hyperaemia** (+=mild) (++=moderate) | **Eyelid dermatitis** | **Lid margin thickening** | **Corneal superficial punctate keratopathy** |  |  |
| **1** | Yes | No | No | Yes | No | No | No | No | 3 | No |
| **2** | Yes | Yes | No | Yes | Yes (+) | No | Yes | No | 6 | No |
| **3** | Yes | No | No | Yes | No | No | No | No | 12 | No |
| **4** | Yes | No | Yes | Yes | No | Yes | No | No | 36 | Yes |
| **5** | Yes | Yes | No | Yes | Yes (+) | No | No | No | 24 | Yes |
| **6** | Yes | No | No | Yes | Yes (+) | No | No | No | 1 | No |
| **7** | Yes | No | No | Yes | No | No | No | Yes | 24 | No |
| **8** | Yes | No | No | Yes | No | No | No | No | 2 | No |
| **9** | Yes | No | No | Yes | Yes (+) | No | No | No | 2 | Yes |
| **10** | Yes | Yes | No | Yes | Yes (+) | Yes | No | No | 3 | Yes |
| **11** | Yes | Yes | No | Yes | Yes (+) | No | No | No | 8 | No |
| **12** | Yes | No | No | Yes | No | No | No | No | 4 | No |
| **13** | No | Yes | No | Yes | Yes (+) | No | No | No | 24 | No |
| **14** | Yes | No | No | Yes | Yes (+) | No | No | No | 18 | No |
| **15** | Yes | Yes | No | Yes | No | No | No | No | 2 | Yes |
| **16** | Yes | No | No | Yes | No | No | No | No | 3 | No |
| **17** | Yes | No | No | Yes | No | No | No | No | 4 | No |
| **18** | Yes | No | No | Yes | No | No | No | No | 2 | No |
| **19** | Yes | No | No | Yes | No | No | No | No | 3 | Yes |
| **20** | Yes | No | No | Yes | No | No | No | No | 8 | Yes |
| **21** | Yes | No | No | Yes | No | Yes | No | No | 2 | No |
| **22** | Yes | Yes | No | Yes | No | No | No | No | 12 | Yes |
| **23** | Yes | Yes | No | Yes | No | No | No | Yes | 9 | No |
| **24** | Yes | Yes | No | Yes | No | No | No | No | 18 | No |
| **25** | Yes | No | No | Yes | Yes (+) | No | No | No | 12 | No |
| **26** | Yes | No | No | Yes | Yes (+) | No | No | No | 5 | No |
| **27** | Yes | Yes | No | Yes | No | No | No | No | 3 | No |
| **28** | Yes | No | No | Yes | No | No | No | No | 3 | No |
| **29** | Yes | No | No | Yes | No | No | No | No | 6 | No |
| **30** | Yes | No | No | Yes | No | No | No | No | 7 | No |
| **31** | Yes | No | No | Yes | No | No | No | No | 2 | No |
| **32** | Yes | No | No | Yes | No | No | No | No | 24 | No |
| **33** | Yes | Yes | No | Yes | No | No | No | No | 5 | No |
| **34** | Yes | No | No | Yes | No | No | No | No | 12 | No |
| **35** | Yes | No | No | Yes | Yes (+) | No | No | No | 4 | No |
| **36** | Yes | No | No | Yes | Yes (+) | No | No | No | 24 | Yes |
| **37** | Yes | No | No | Yes | No | No | No | No | 9 | Yes |
| **38** | Yes | Yes | No | Yes | No | No | No | No | 12 | No |
| **39** | Yes | No | No | Yes | No | No | No | No | 16 | No |
| **40** | Yes | No | No | Yes | Yes (+) | No | No | No | 2 | Yes |
| **41** | Yes | Yes | No | Yes | Yes(+) | No | No | No | 6 | No |
| **42** | Yes | No | No | Yes | No | No | No | No | 18 | No |
| **43** | Yes | No | No | Yes | No | No | No | No | 3 | No |
| **44** | Yes | Yes | No | Yes | Yes (+) | No | No | No | 3 | No |
| **45** | Yes | No | No | Yes | No | No | No | No | 12 | No |
| **46** | Yes | Yes | No | Yes | Yes (+) | No | No | No | 3 | No |
| **47** | Yes | No | No | Yes | No | No | No | No | 3 | No |
| **48** | Yes | Yes | No | Yes | Yes (+) | No | Yes | No | 10 | Yes |
| **49** | Yes | No | No | Yes | No | No | No | No | 5 | No |
| **50** | Yes | Yes | No | Yes | No | No | No | No | 12 | No |
| **51** | Yes | No | No | Yes | No | No | No | No | 24 | Yes |
| **52** | Yes | Yes | No | Yes | Yes (+) | No | No | No | 14 | Yes |
| **53** | Yes | Yes | No | Yes | No | No | No | Yes | 6 | No |
| **54** | Yes | No | No | Yes | No | No | No | No | 8 | No |
| **55** | Yes | No | No | Yes | No | No | No | No | 4 | No |
|  | All patients except 1 |  |  | All patients | 32% of patients |  |  |  | 9 months average |  |
