# Supplementary material for: Chronic tarsal conjunctivitis
Source: BMC Ophthalmol. 2016 Jul 29;16:130. doi: 10.1186/s12886-016-0294-1 (PMC4965890; doi:10.1186/s12886-016-0294-1)
Supplement: Additional file 2: Table S2. — Outcome of 55 patients with Chronic Tarsal Conjunctivitis, as of April 2015. (DOCX 26 kb) [file 12886_2016_294_MOESM2_ESM.docx]

**Table 2:** Outcome of 55 patients with Chronic Tarsal Conjunctivitis, as of April 2015

| **Patient ID** | **Outcome (Yes/No)** | | | **Total duration of symptoms (months)** | **Patch test results**  No= negative  Blank = not available |
| --- | --- | --- | --- | --- | --- |
|  | **Referred to Hospital Eye Service** | **Resolution of condition** | |  |  |
|  |  | **Lost to follow up, resolution assumed** | **Resolution known** |  |  |
| **1** | Yes | No | Yes | 18 |  |
| **2** | Yes | No | Yes | 6 |  |
| **3** | Yes | No | Yes | 60 |  |
| **4** | No | No | Yes | 30 |  |
| **5** | No | No | Yes | 48 | No |
| **6** | No | No | Yes | 6 | No |
| **7** | No | No | Yes | 30 |  |
| **8** | Yes | No | Yes | 9 | +ve  (Nickel) |
| **9** | No | No | Yes | 22 | No |
| **10** | No | No | Yes | 21 | No |
| **11** | No | No | Yes | 20 | +ve  (Nickel) |
| **12** | No | No | Yes | 10 |  |
| **13** | No | Yes | No | 36 |  |
| **14** | No | No | Yes | 25 | No |
| **15** | No | No | Yes | 16 | +ve  (Nickel) |
| **16** | No | No | Yes | 15 |  |
| **17** | No | No | Yes | 16 |  |
| **18** | No | No | Yes | 24 |  |
| **19** | No | No | Yes | 28 |  |
| **20** | No | No | Yes | 18 | +ve  (Nickel) |
| **21** | Yes | Yes | No | 10 |  |
| **22** | No | No | Yes | 18 |  |
| **23** | No | No | Yes | 17 | +ve  (Potassium dichromate) |
| **24** | No | Yes | No | 21 |  |
| **25** | No | No | Yes | 16 | +ve  (Nickel) |
| **26** | No | Yes | No | 8 |  |
| **27** | No | No | Yes | 7 | +ve  (MI,0.2%) |
| **28** | No | Yes | Yes | 18 |  |
| **29** | No | No | Yes | 10 |  |
| **30** | No | Yes | No | 11 |  |
| **31** | No | No | Yes | 14 |  |
| **32** | No | No | Yes | 29 |  |
| **33** | No | No | Yes | 9 |  |
| **34** | No | No | Yes | 15 |  |
| **35** | Yes | No | Yes | 30 | +ve  (Nickel,PPD) |
| **36** | No | No | Yes | 42 | No |
| **37** | No | No | Yes | 13 | No |
| **38** | No | No | Yes | 26 | No |
| **39** | No | No | Yes | 28 | +ve  (Nickel) |
| **40** | No | Yes | No | 7 |  |
| **41** | No | No | Yes | 12 | +ve  (Nickel) |
| **42** | No | No | Yes | 22 | +ve  (fragrance mix1,balsam of Peru) |
| **43** | No | No | Yes | 8 |  |
| **44** | Yes | No | Yes | 22 |  |
| **45** | No | Yes | No | 15 |  |
| **46** | Yes | Yes | No | 10 |  |
| **47** | No | No | Yes | 15 |  |
| **48** | No | No | Yes | 24 | No |
| **49** | No | No | Yes | 7 |  |
| **50** | No | No | Yes | 15 |  |
| **51** | No | No | Yes | 30 | No |
| **52** | No | Yes | Yes | 22 | +ve(Nickel) |
| **53** | No | No | Yes | 14 | +ve(Nickel) |
| **54** | No | Yes | No | 11 |  |
| **55** | No | Yes | Yes | 7 |  |
